# A novel scatterplot-based method to detect copy number variation (CNV)

**DOI:** 10.3389/fgene.2023.1166972

**Published:** 2023-07-06

**Authors:** Jia-Lu Qiao, Rebecca T. Levinson, Bowang Chen, Stefan T. Engelter, Philipp Erhart, Brady J. Gaynor, Patrick F. McArdle, Kristina Schlicht, Michael Krawczak, Martin Stenman, Arne G. Lindgren, John W. Cole, Caspar Grond-Ginsbach

**Affiliations:** ^1^Department of Vascular and Endovascular Surgery, University Hospital Heidelberg, Heidelberg, Germany; ^2^ Institute for Computational Biomedicine, Faculty of Medicine, Heidelberg University Hospital, Heidelberg, Germany; ^3^ Department of General Internal Medicine and Psychosomatics, University Hospital Heidelberg, Heidelberg, Germany; ^4^ National Center for Cardiovascular Diseases, Beijing, China; ^5^ Neurorehabilitation Unit, University of Basel and University Center for Medicine of Aging Felix Platter Hospital, Basel, Switzerland; ^6^ Department of Medicine, University of Maryland School of Medicine, Baltimore, MD, United States; ^7^ Institute of Diabetes and Clinical Metabolic Research, University Medical Center Schleswig-Holstein, Kiel, Germany; ^8^ Institute of Medical Informatics and Statistics, Kiel University Medical Center Schleswig-Holstein, Kiel, Germany; ^9^ Department of Clinical Sciences Lund, Lund University, Skåne University Hospital, Lund, Sweden; ^10^Department of Neurology, Lund University, Skåne University Hospital, Lund, Sweden; ^11^ Veterans Affairs Maryland Healthcare System, University of Maryland School of Medicine, Baltimore, MD, United States

**Keywords:** copy number variation (CNV), pennCNV, scatterplot, filtering, quality control

## Abstract

**Objective:** Most methods to detect copy number variation (CNV) have high false positive rates, especially for small CNVs and in real-life samples from clinical studies. In this study, we explored a novel scatterplot-based method to detect CNVs in microarray samples.

**Methods:** Illumina SNP microarray data from 13,254 individuals were analyzed with scatterplots and by PennCNV. The data were analyzed without the prior exclusion of low-quality samples. For CNV scatterplot visualization, the median signal intensity of all SNPs located within a CNV region was plotted against the median signal intensity of the flanking genomic region. Since CNV causes loss or gain of signal intensities, carriers of different CNV alleles pop up in clusters. Moreover, SNPs within a deletion are not heterozygous, whereas heterozygous SNPs within a duplication show typical 1:2 signal distribution between the alleles. Scatterplot-based CNV calls were compared with standard results of PennCNV analysis. All discordant calls as well as a random selection of 100 concordant calls were individually analyzed by visual inspection after noise-reduction.

**Results:** An algorithm for the automated scatterplot visualization of CNVs was developed and used to analyze six known CNV regions. Use of scatterplots and PennCNV yielded 1019 concordant and 108 discordant CNV calls. All concordant calls were evaluated as true CNV-findings. Among the 108 discordant calls, 7 were false positive findings by the scatterplot method, 80 were PennCNV false positives, and 21 were true CNVs detected by the scatterplot method, but missed by PennCNV (i.e., false negative findings).

**Conclusion:** CNV visualization by scatterplots allows for a reliable and rapid detection of CNVs in large studies. This novel method may thus be used both to confirm the results of genome-wide CNV detection software and to identify known CNVs in hitherto untyped samples.

## Introduction

Copy number variation (CNV) is defined as the genomic presence of a given DNA sequence element >50 bp in copy number different from a reference genome. CNVs are a common type of structural variation in eukaryotic genomes, and single nucleotide polymorphism (SNP) microarray technology enables high-throughput, genome-wide detection of CNVs in many species, including humans. However, due to high noise-to-signal ratios, inter-sample variability and technical differences between microarray platforms, reliable detection of CNVs remains challenging (Carter, 2007; Grond-Ginsbach et al., 2018). Most current methods of CNV detection have unacceptably high false positive rates, in particular for small CNVs (i.e., CNVs covered by fewer than 20 SNPs) (Lin et al., 2011). One popular way to overcome these limitations has been manual expert review of the original CNV calls, aimed at filtering-out false positives before further downstream analysis, or experimental validation (Ginsbach et al., 2013; Grond-Ginsbach et al., 2017; Glessner et al., 2021). In the present study, we developed a novel scatterplot-based method drawing upon the visualization of SNP signal intensities to detect individual CNVs. The method exploits the observation that samples with duplication or deletion alleles appear as satellite clusters with increased or decreased signal intensities, respectively, in scatterplots. The characteristic distribution of the B-allele frequency (BAF) in samples with a copy number (CN) between 0 and 4 was used to confirm CNVs called from satellite clusters in the corresponding scatterplots.

## Materials and methods

Illumina 2.5 M SNP data from 13,245 individuals from the Health and Retirement study (Aschwanden et al., 2019), genotyped at the Center of Inherited Disease Research (CIDR) of the Johns Hopkins University School of Medicine, Baltimore, United States, were used for analysis. The study population is part of the Copy Number Variation and Stroke (CaNVAS) study (Cole et al., 2021). CNV analysis was also performed in all samples using the PennCNV software as described before (Wang et al., 2007; Grond-Ginsbach et al., 2017).

Six genomic regions with 10 genic CNVs were chosen for visualization with scatterplots (Table 1). The CNVs were selected in such a way that 1. The CNV-region included at least one protein-coding gene, 2. The minor allele count was not too low and 3. That all observed variants in the region had similar or identical size. All selected CNVs have been reported previously in scientific publications (Edsgärd et al., 2011; Tse et al., 2011; Bertelsen et al., 2016; Crawford et al., 2019) or were listed as gold standard variants in the DGV database of human structural variants. For each CNV region, the median signal intensities (Log R Ratios, LRR) of the SNPs located within the region and within both flanking regions were calculated for each individual sample. CNV target and flanking regions were chosen to be similar in size and to contain at least 30 SNPs. Scatterplots were created by plotting the sample-specific median signal intensity of the target CNV region (*x*-axis) against that of the 5′- or 3′-flanking regions (*y*-axis). Information about the B-allele frequencies (BAF) of SNPs located within the target region was used for further CNV characterization. Three different metrics were calculated to this end for each sample, namely, 1) the total number of homozygous SNPs (defined as BAF<0.01 or BAF>0.99), 2) the total number of di-allelic heterozygous SNPs (defined as 0.47<BAF<0.53), and 3) the total number of tri-allelic heterozygous SNPs (defined as 0.3<BAF<0.36 or 0.63<BAF<0.69). Finally, the difference Δ between the median SNP signal intensity within the target and each flanking region was calculated for each CNV region and sample. Supplementary file 1 and 2 give a more detailed explanation of the scatterplot tool as well as an instruction for use and some more examples of application. A python code for the scatterplot-based calculations, a datafile for the CNV regions analyzed in this study, and an instruction for use are available at https://github.com/thelevinsonlab/CaNVAS_CNV.

**TABLE 1:** CNV regions studied with PennCNV, scatterplots and subsequent visual inspection after noise reduction.

CNV region	Chr2:110	Chr 3:151	Chr6:29	Chr6:31	Chr9:5	Chr17:33	Total
5′breakpoint (hg19)	2:110852875	3:151514590	6:29096414	6:31360225	9:5304710	17:33684035	
3′breakpoint (hg19)	2:110942946	3:151546695	6:29161435	6:31451680	9:5337760	17:33768199	
No. SNPs	97	33	58	241	27	93	
Protein-coding genes	*MALL, NPHP1, MTLN*	*AADAC*	*OR2J2*	*MICA, HCP5*	*RLN2, RLN1*	*SLFN11, SLFN12, SLFN13*	
Human DGVa ID	esv3591950	esv3598188			esv3619374		
No. Duplications							
True	99	0	20	45	103	0	267
False negatives							
*PennCNV*	3	0	1	0	0	0	4
*Scatterplot*	0	0	0	0	0	0	0
False positives							
*PennCNV*	0	0	0	0	1	0	1
*Scatterplot*	0	0	1	0	2	0	3
No. Deletions							
True	69	262	36	199	154	53	773
False negatives							
*PennCNV*	1	11	1	0	4	0	17
*Scatterplot*	0	0	0	0	0	0	0
False positives							
*PennCNV*	57	3	5	8	0	6	79
*Scatterplot*	0	3	0	0	1	0	4

In the CaNVAS HRS sample (*n* = 13,524), six CNV regions were analyzed with PennCNV and with scatterplots. The results visualized were obtained after noise reduction with the ‘noise-free-cnv’ software for CNV validation.

Human DGVa-ID: identifier from the Human DGVa Structural Variation database; true: CNV call confirmed by visual inspection after noise-reduction; false: CNV call that could not be confirmed by visual inspection after noise-reduction.

Separate satellite clusters that comprised samples of increased or decreased signal intensity were interpreted as harboring duplication or deletion alleles. We did not use predefined cut-off levels to discriminate between carriers of a normal (Copy Number = 2) CNV region and those with a deletion or a duplication. Instead, the scatterplot script produced clearly separated clusters for each analyzed CNV region. Subsequently, individuals were genotyped for this specific CNV region on the basis of the observed pattern of clustering. For further validation of an identified satellite cluster, Δ was plotted against the number of either di-allelic or tri-allelic heterozygous SNPs.

Scatterplot-based findings made in the six selected CNV regions were compared to PennCNV calls. A PennCNV call was considered ‘positive’ for a given CNV if it overlapped the target region of the CNV by at least 50%, but was no larger than twice that region. For the validation of both PennCNV and scatterplot-based findings, the microarray data were analyzed with the ‘noise-free-cnv’ software. A CNV call was considered a true positive finding when was validated by visual inspection as described (Ginsbach et al., 2013).

Sample-wise quality control of the SNP-microarray data was performed with three different metrics: 1) the total number of PennCNV calls per sample, 2) the variance of all autosomal LRR values per sample, and 3) the percentage of successfully genotyped SNPs (“call rate”) per sample. Samples were ranked according to each quality metric and labelled better (i.e., fewer (i.e., non-outlier number of) PennCNV calls, lower variance, or higher call rate) than x% of all analyzed samples.

## Results

Six CNV regions were analyzed with two different CNV calling methods in 13,254 high-density SNP microarray datasets (Table 1). All regions studied contained protein-coding genes. Both deletion and duplication alleles were found in four of the six regions, two regions contained only deletions as B-alleles.

**FIGURE 1:**
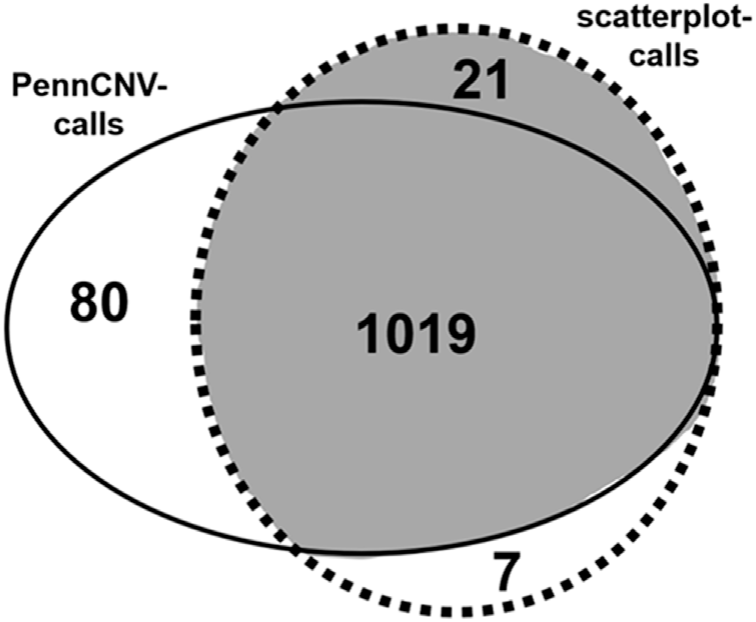
CNV calling with PennCNV and scatterplot analysis. Venn-diagram of PennCNV calls (uninterrupted line) and scatterplot-based CNV calls (dotted line). CNV calls considered true findings after visual inspection are highlighted in grey.

Analysis with PennCNV software yielded 1099 CNV calls whilst scatterplot visualization suggested the presence of 1047 CNVs in all regions combined (Figure 1). Of the total of 1127 CNV findings, 1019 were consistently identified with both PennCNV and scatterplot analysis. The remaining 108 CNV calls were discordant in the sense that they were made with only one of the two tools.

Visual inspection of the 108 discordant calls and of 100 randomly selected concordant calls revealed that all concordant calls were most likely true CNVs. Of the 108 discordant calls, 80 were PennCNV false positives and 7 were scatterplot false positives (Figure 1). The remaining 21 discordant calls were true CNVs detected with scatterplots, but not PennCNV. These calls thus represented PennCNV false negatives; no false negative calls were made with scatterplots (Figure 1). False findings were observed in each of the six analyzed CNV-regions. Most false PennCNV-findings were false-positive deletions in region 2:110852875-110942946.

**FIGURE 2:**
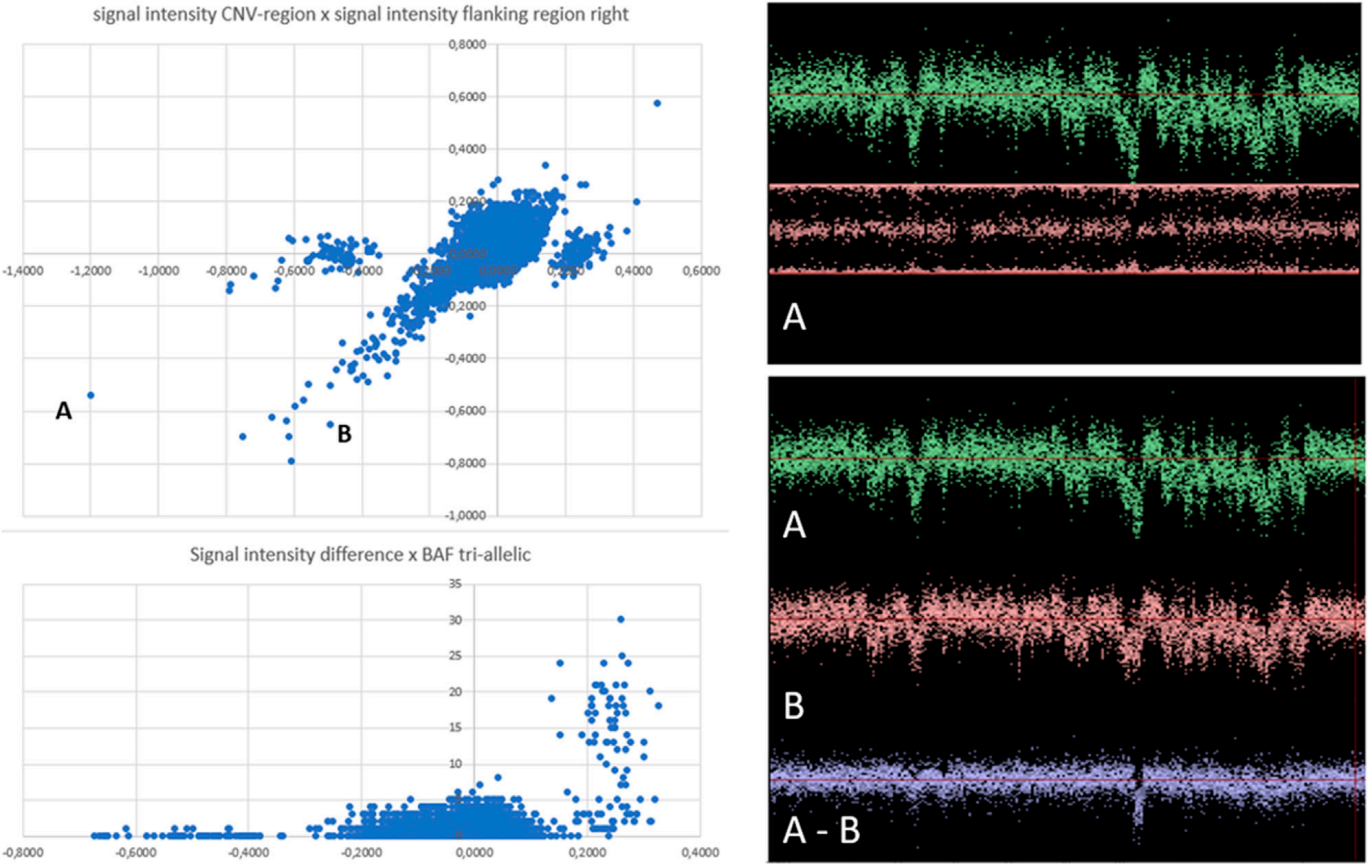
Scatterplot visualization and subsequent visual inspection of individual findings. Left panel: Scatterplots of CNV region chr2:110852875-110942946. Every dot corresponds to one of 13,254 analyzed microarray SNP datasets. In the upper left panel, each dot represents the signal intensity within the CNV-region (x-axis), plotted against the signal intensity of the left flanking region (y-axis). Three separate cluster appear. The central cluster is centered in both axes around zero, suggesting “normal” signal strength in the CNV-region as well as in the flanking region for all individuals in this cluster. Outlier dots of this cluster follow a diagonal pattern, indicating microarrays with reduced or increased signal intensities in this region in both the CNV region and in the flanking region. The outlier dots apparently represent microarrays with strong genomic waves, affecting both the CNV and the flanking regions. Two individuals with particularly strong waves, but apparently belonging to different clusters, were labelled “A” and “B” and will be individually visualized in a second step, as shown in the right panels. Dots in the satellite clusters represent microarrays with signal intensities that differ between the of CNV-region and flanking region. In the left cluster, the CNV region has lower signal intensity (the cluster centers around −0.5). In the right cluster, dots represent samples with higher signal intensity in the CNV (values around +0,25) compared to the flanking region (baseline values around 0). Signal intensities of SNPs of a large region of chromosome 2 of two individual microarray samples were shown in the right panels. The visualized genomic region includes CNV-region chr2:110852875-110942946. The selected individuals (sample A and sample B) were represented in the clusterplots at the left side of panel by dots A and B. The low signal intensities of the flanking regions of both individuals (negative values of y-axis) corresponds to the strong wave pattern seen of the signal intensities int he right side panels. This strong waves may falsely suggest the presence of duplications (strong signal across a region) or deletions (weak signals). The pattern of genomic waves in sample A and sample B seems similar. However, subtraction of signal intensities of sample A and B **(A-B)** reveaed that individual A and individual B have different signal intensities in CNV region chr2:110852875-110942946, as inferred from the scatterplots.

Overall, 1040 true CNVs comprising 267 duplication and 773 deletion B-alleles were recorded in the six regions studied (Table 1). Figure 2 exemplifies the scatterplot-based calling of the CNV region on chromosome 2. It also highlights the validation of a suggestive deletion that was not identified with PennCNV. After noise-reduction of the LRR values by subtraction of LRR values of another sample with similar genomic waves (Figure 2), this deletion was confirmed to be a true finding. Supplementary file 3 show scatterplots in different study populations and analyzes one example of a CNV in a region of clonal mosaicism.

**FIGURE 3:**
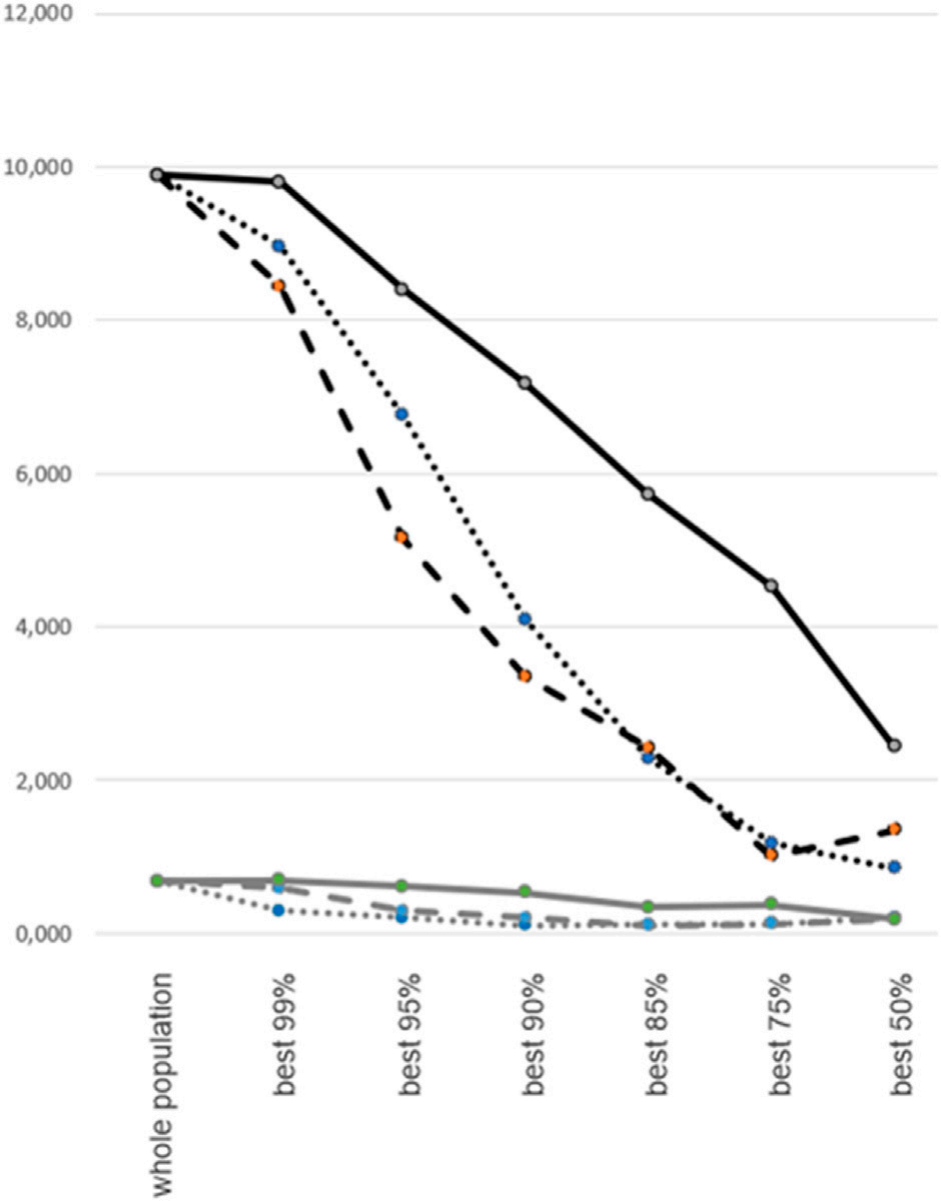
Rate of false CNV calls after case-level quality control filtering. Percentage of false CNV calls by PennCNV (black lines) and scatterplot-based analysis (gray lines). False call rates (represented on the y-axis) were calculated for the whole study dataset and for subsets following the exclusion of 1%, 5%, 10%, 15%, 25% or 50% of low-quality samples (x-axis). Quality control filtering was based upon variance of LRR values (interrupted lines), number of outlier PennCNV calls (dotted lines), or SNP genotyping call rate (continuous lines).

Since quality control (QC) filtering and the exclusion of low-quality samples is common practice prior to PennCNV analysis, we assessed the performance of PennCNV and the novel scatterplot-based method after QC filtering steps of varying stringency (Figure 3). The ratio of false-to-true CNV findings was calculated for the whole study dataset (n = 13,254) and for subsets resulting from the exclusion of 1%, 5%, 10%, 15%, 25%, and 50%, respectively, of low-quality samples. The latter were defined by 1) a high (“outlier”) number of PennCNV calls, 2) a high variance of LRR values, or 3) a low SNP call rate. The ratio of false-to-true CNV findings was consistently found to be about 10-fold higher for PennCNV than for the scatterplot-based method. QC filtering by the exclusion of samples with high LRR variance reduced the rate of false findings most efficiently, followed by an exclusion based upon an outlier number of PennCNV calls. Exclusion of samples with low SNP call rate had the smallest effect upon the quality of CNV calling.

## Discussion

The present study has two key findings: 1) Visualization of CNV regions by signal intensity scatterplots allows a rapid validation of PennCNV calls, even in large study populations, and 2) compared to PennCNV, scatterplot-based CNV calling has lower false positive and negative rates, even without the prior exclusion of low-quality samples from further analysis. In the large dataset analyzed here, PennCNV yielded about 10% false positive and negative findings combined whilst the error rate of scatterplots was around 1%. Our study also revealed that the exclusion of low-quality samples results in a significant reduction of the error rate mostly for PennCNV, and less so for the scatterplot-based approach. Overall, it may thus be concluded that the use of scatterplots not only allows rapid and reliable validation of PennCNV calls, but also enables the extension of CNV analyses to low-quality cases which would otherwise be excluded. Finally, scatterplots may help to identify some PennCNV findings in discovery samples as false positives, and may identify additional true CNVs that went undetected by PennCNV.

The above notwithstanding, our study also has some limitations. Only a small number of known CNV regions were covered. The performance of the scatterplot analysis across different SNP-microarray platforms and in cases with clonal mosaicism was only illustrated in a supplementary file and not analyzed in depth. CNV-regions close to the centromeres or telomeres may be subject to very strong genomic waves and may therefore be enriched for false-positive PennCNV deletions. None of the CNV-regions analyzed in this study was located in subtelomeric or pericentric regions. We nevertheless observed heterogeneity across the analyzed CNV regions with the majority of the false-positive PennCNV findings occurring in a single region. Since this clearly gives our current work the character of a pilot study, we are planning a follow-up study to analyze a larger set of syndromic CNVs (Wang et al., 2007) in samples from the CaNVAS study (Cole et al., 2021), genotyped on different platforms.

Visual inspection of discordant CNV calls, both with or without prior reduction of systemic noise, allowed us to distinguish between true and false positive findings. The final set of confirmed true CNVs was then used as a ‘gold standard’ for the comparative evaluation of the false positive and negative rates of PennCNV and of our scatterplot-based method of CNV detection. Since CNVs that were not recognized by either tool were not considered, this *post hoc* evaluation did not allow estimation of the underlying sensitivity and specificity values of the two approaches.

It is important to underline some fundamental differences between tools that were developed for the genome-wide detection of any potential CNV (like PennCNV) and the current scatterplot method that is a tool for the analysis of CNV in a specific, predefined genomic region. Whereas PennCNV allows the identification of an infinite number of different CNVs, the scatterplot tool discriminates between a very limited number of copy number states within a single genomic locus. As a consequence, there is an innumerable set of potential false PennCNV findings, whereas the number of potential false scatterplot findings is small. Another important difference between both strategies is that scatterplots of very large study populations show particularly clear clusters, whereas PennCNV analysis of very large study population becomes extremely laborious. Whereas the PennCNV tool was developed for the genome-wide analysis of single individuals one after another, the scatterplot tool was developed for the genotyping of a single CNV-locus simultaneously in a large study population.

CNV calling with the aid of scatterplots has several advantages. Similar to SNP genotyping (Lovmar et al., 2005; Schillert et al., 2009), visualization of CNVs provides an easily comprehensible and transparent means to identify carriers of variant alleles and also of instances of unsuccessful genotyping. The clear separation of CNV genotype clusters, or their partial overlap, clearly corresponds to the straightforwardness, or other, of a particular CNV genotype. Noisy CNV scatterplot clusters may also point towards a variety of distorting factors, including the presence of multiple variants of different size, the choice of false CNV region breakpoints, CNVs within the flanking regions, or low quality of the underlying microarray SNP data. Notable in this context, one strength of the scatterplot-based approach is its ability to obviate the need for stringent exclusion of low-quality cases from analysis. This not only increases the ability to detect rare CNVs in large study samples, but high-quality SNP genotyping must not necessarily remain the only suitability criterion for CNV analysis. Our results also demonstrated that PennCNV yields false positive and negative findings at considerable rates, again predominantly in low-quality cases. Finally, we observed that QC filtering based upon LRR variance may be at least as efficient as QC filtering based upon outlier numbers of PennCNV calls.

In conclusion, our study demonstrates that the use of scatterplots represents an efficient tool for both CNV visualization and validation. The method could be incorporated into existing analysis pipelines to evaluate CNVs called *ab initio* by other, automated detection algorithms, or may be used for the targeted analysis of known pathogenically relevant CNV regions in large study populations.

## Data Availability

The data analyzed in this study is subject to the following licenses/restrictions: In this analysis, we used SNP-microarry dataset from the CaNVAS study. Requests to access these datasets should be directed to JC, JCole@som.umaryland.edu.

## Supplementary material

A python code for the scatterplot-based calculations, a datafile for the CNV regions analyzed in this study, and an instruction for use are available at https://github.com/thelevinsonlab/CaNVAS_CNV.
