# Supplementary material for: A novel scatterplot-based method to detect copy number variation (CNV)
Source: Front Genet. 2023 Jul 6;14:1166972. doi: 10.3389/fgene.2023.1166972 (PMC10359988; doi:10.3389/fgene.2023.1166972)
Supplement: Supplementary file 1 [file DataSheet1.docx]

**Supplementary file 1.**

The CaNVAS CNV script allows the detection of CNV in large SNP-microarray samples. The script is based on a list of observed CNVs. In CaNVAS, PennCNV-findings are used potential CNV and can be confirmed as “true findings” with the aid of the current algorithm.

The CaNVAS CNV algorithm performs for each microarray sample the following steps:

1. For each CNV-region, all SNPs within the CNV region are identified. For reference, SNPs in two flanking genomic regions (5’end as well as 3’ end) will also be defined. A list of CNV-areas for the six CNV-regions analyzed in the current study was shown as Supplementary file 2.

2. The mean and the median signal intensities (“Log R ratio, LRR” values) of the SNPs within the CNV region and of the flanking regions are calculated.

3. The SNPs of the CNV-region are classified according to their genotypes (B-allele frequencies, BAF) into 3 groups: homozygous SNPs (BAF<0.01 or BAF >0.99), heterozygous SNPs with 2 alleles (0.47<BAF<0.53), or heterozygous SNPs with 3 alleles (either 0.30<BAF<0.36 or 0.63<BAF<69). NB: Some SNPs may remain unclassified.

An output file is produced with the following data:

1. Name of the microarray samples

2. Mean and median LRRvalues of CNV target region and of both flanking regions.

3. Number of SNPs that are homozygous, bi-allelic heterozygous or tri-alleles heterozygous.

The data of output file can be visualized by the attached plotter script. After transfer of the data into an excel file, multiple scatterplots can be created by hand.

The following figures show the output file for a CNV regions on chromosome 19 (region 19_53325198_53358889) in the population of the Health Retirement Study. Columns C-M show mean and median intensities of target region (C + E) and flanking regions. This CNV-regions is also included in the list of CNV-regions shown below (1.5).

Plots of the median target region and the median left flanking regions show three well separated clusters. The large cluster in the center are the “normal” (2-Copy) samples. The satellite clusters represent the cases with deletions (left side) and duplications. Figure 3 justifies the interpretation of the right-sided cluster as duplication-cases.

Calculation of the difference between the intensities of the CNV target region and a flanking region may facilitate the CNV-genotyping, in particular after plotting of these differences against the number of disomic heterozygous or the number of trisomic heterozygous SNPs.


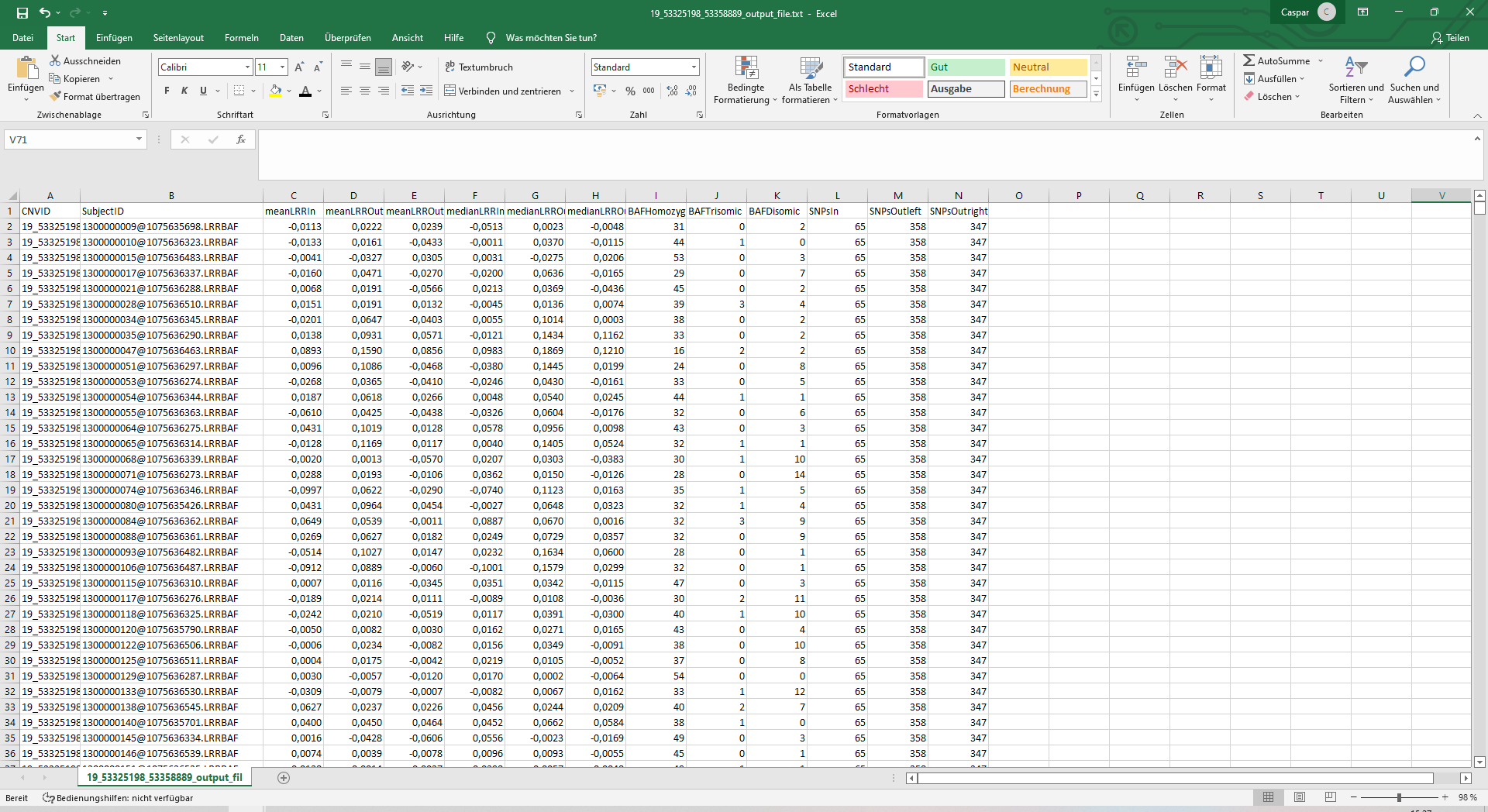


For final evaluation of a CNV-finding, we usually inspect the individual microarray sample. For this we use the noise-free-CNV software ([GitHub - ginsbach/noise-free-cnv: Program for analyzing and manipulating DNA microarray data](https://github.com/ginsbach/noise-free-cnv)).


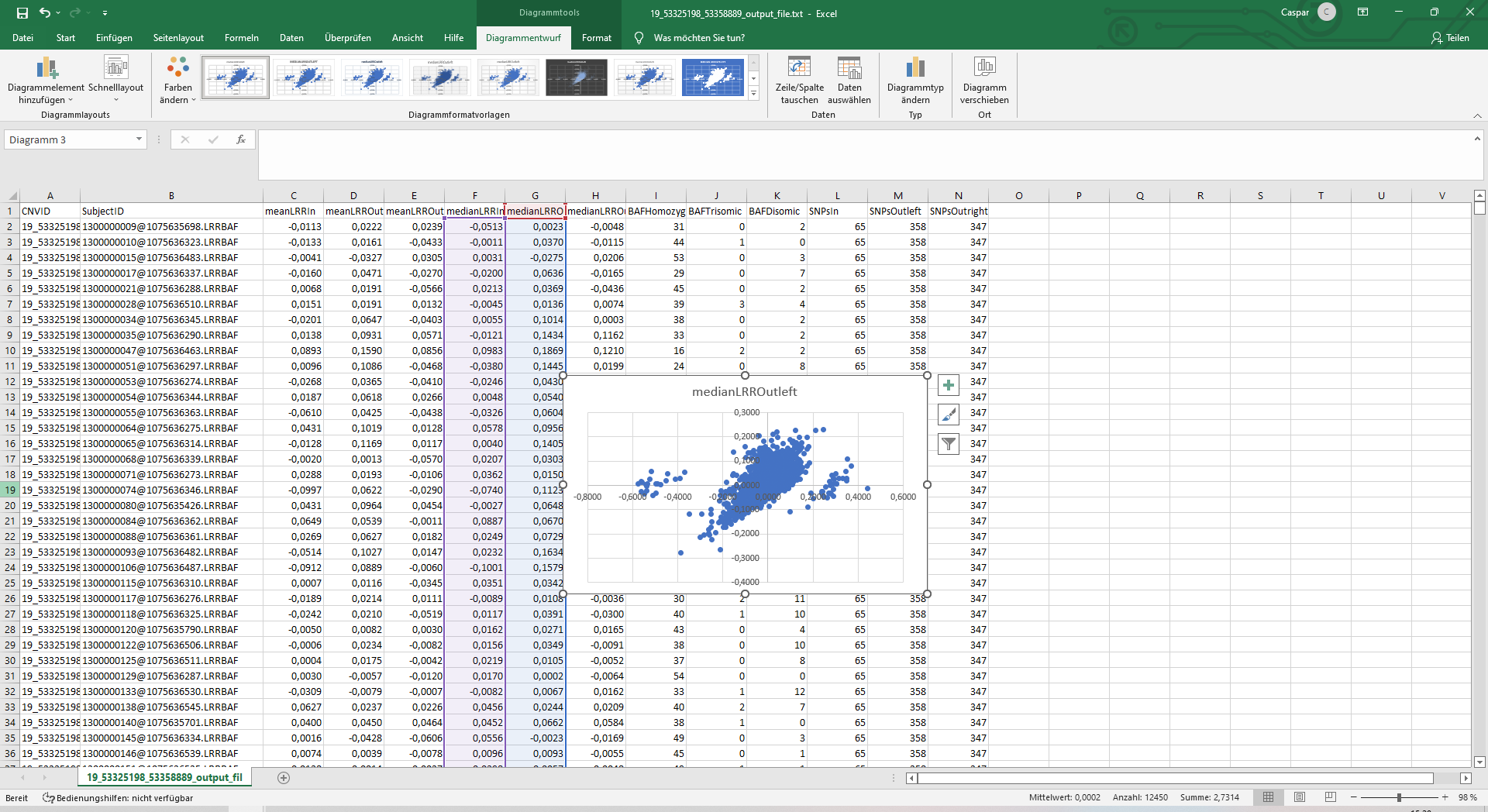


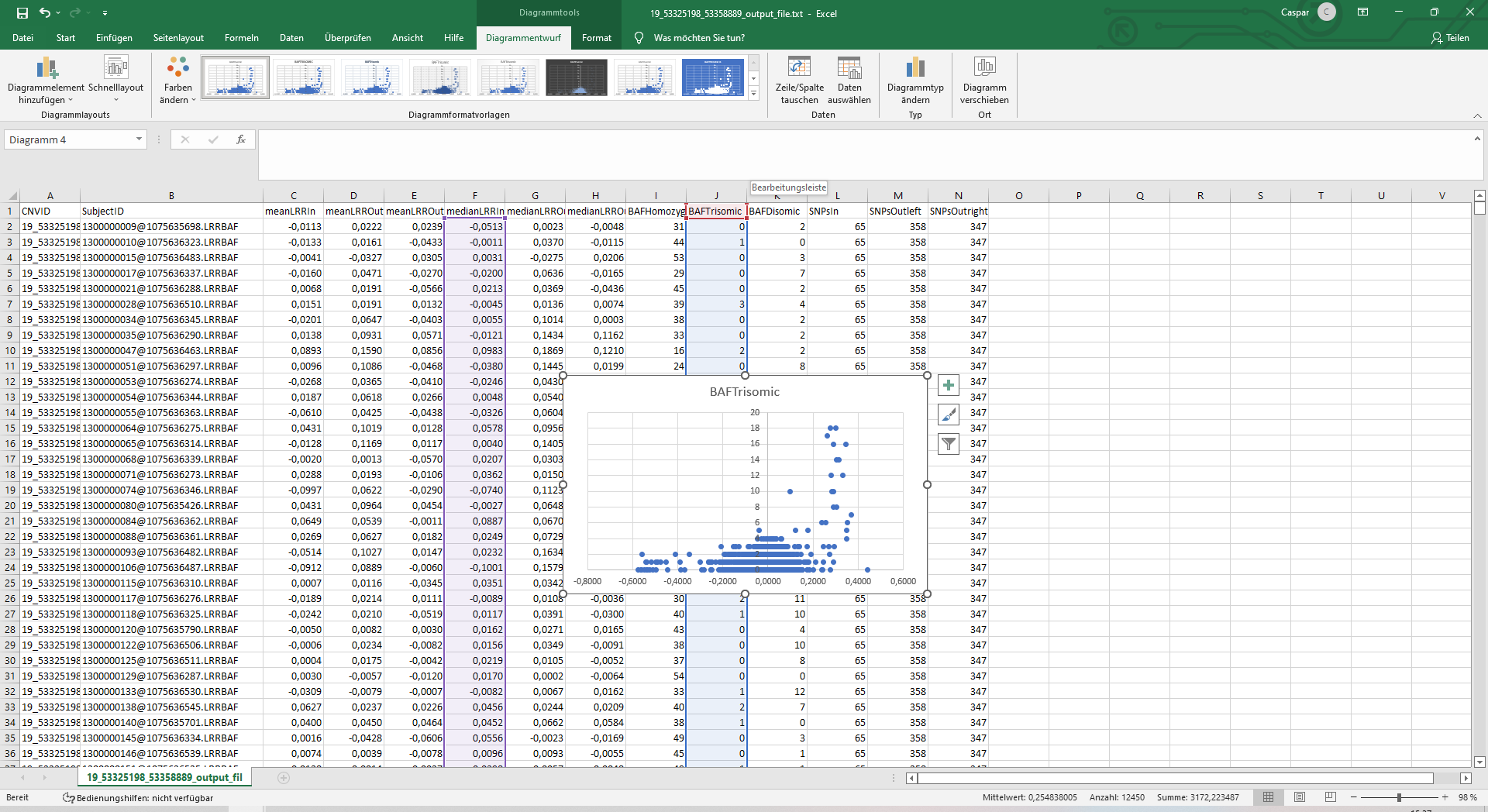


**Supplementary file 2.**


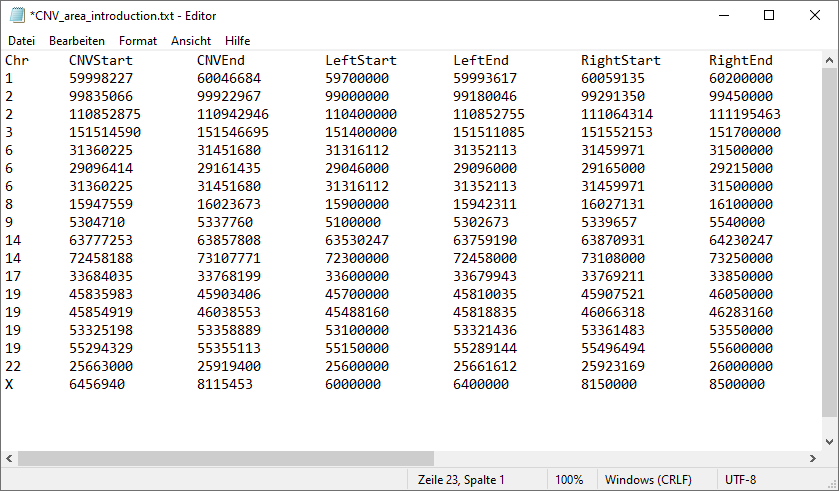


**Supplementary File 3.**

1. Analyses of CNV regions across three different platforms


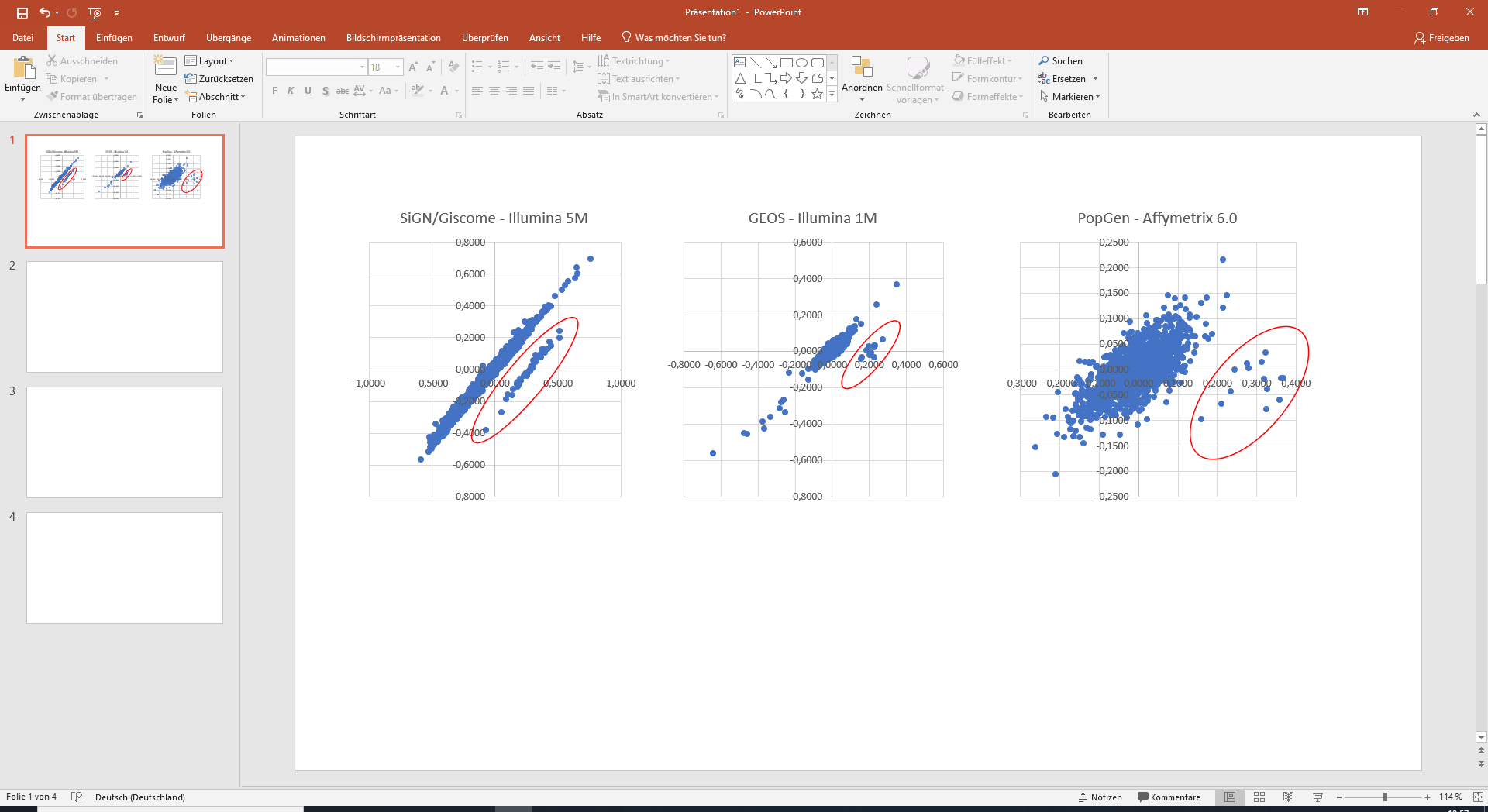


Signal intensity scatterplots of CNV-region (chromosome 9:138147348-138308955) in three CaNVAS cohorts (Sign/Giscome, n= 5870, 422 SNPs within CNV-region; GEOS, n=1444, 87 SNPs within CNV-region; and PopGen, n=1262, 115 SNPs within CNV-region). For detailed information about these study cohorts see Cole et al. (reference 8 of manuscript). Each study population was analyzed on a different platform. Cases with a duplication in this CNV region were encircled. For confirmation of the duplication, first the difference $(\Delta)$between the mean signal intensities of target and flanking regions was calculated and subsequently, BAF-category counts were plotted against $\Delta(see below)$.


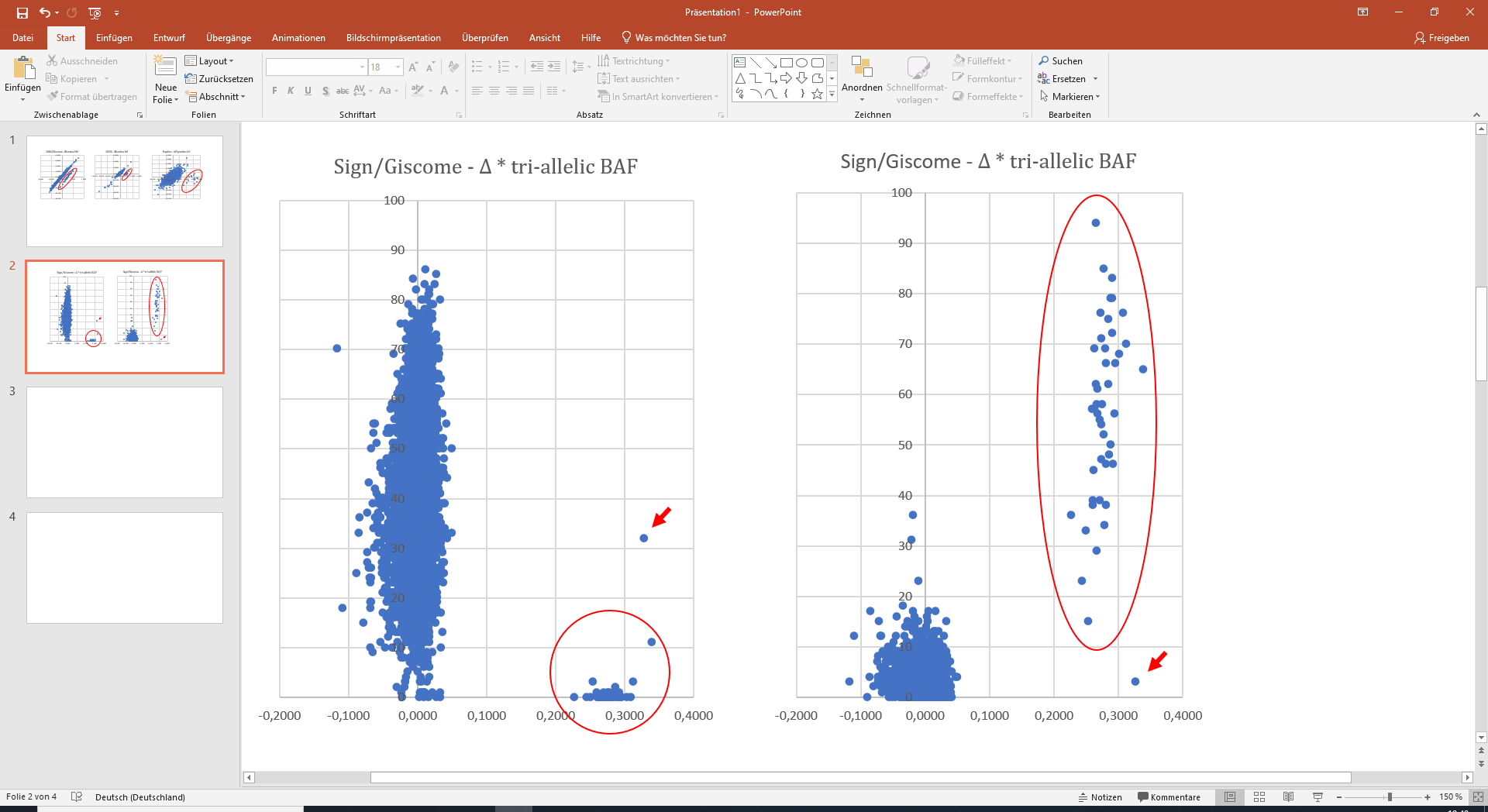


Sign/Giscome sample: Scatterplots of Δ versus the count of bi-allelic (left panel) and tri-allelic (right panel) BAF-values. Delta values of encircled cases suggests CN=3 (signal in CNV region is higher than in flanking SNP). Low count of di-allelic SNPs and high count of tri-allelic SNPs confirms CNV-calling. One single outlier point (red arrows) represents a case of unclear genotype – possibly CN=4. Visualization of this individual case in noise-free-CNV software is mandatory (references 5,6 of manuscript).


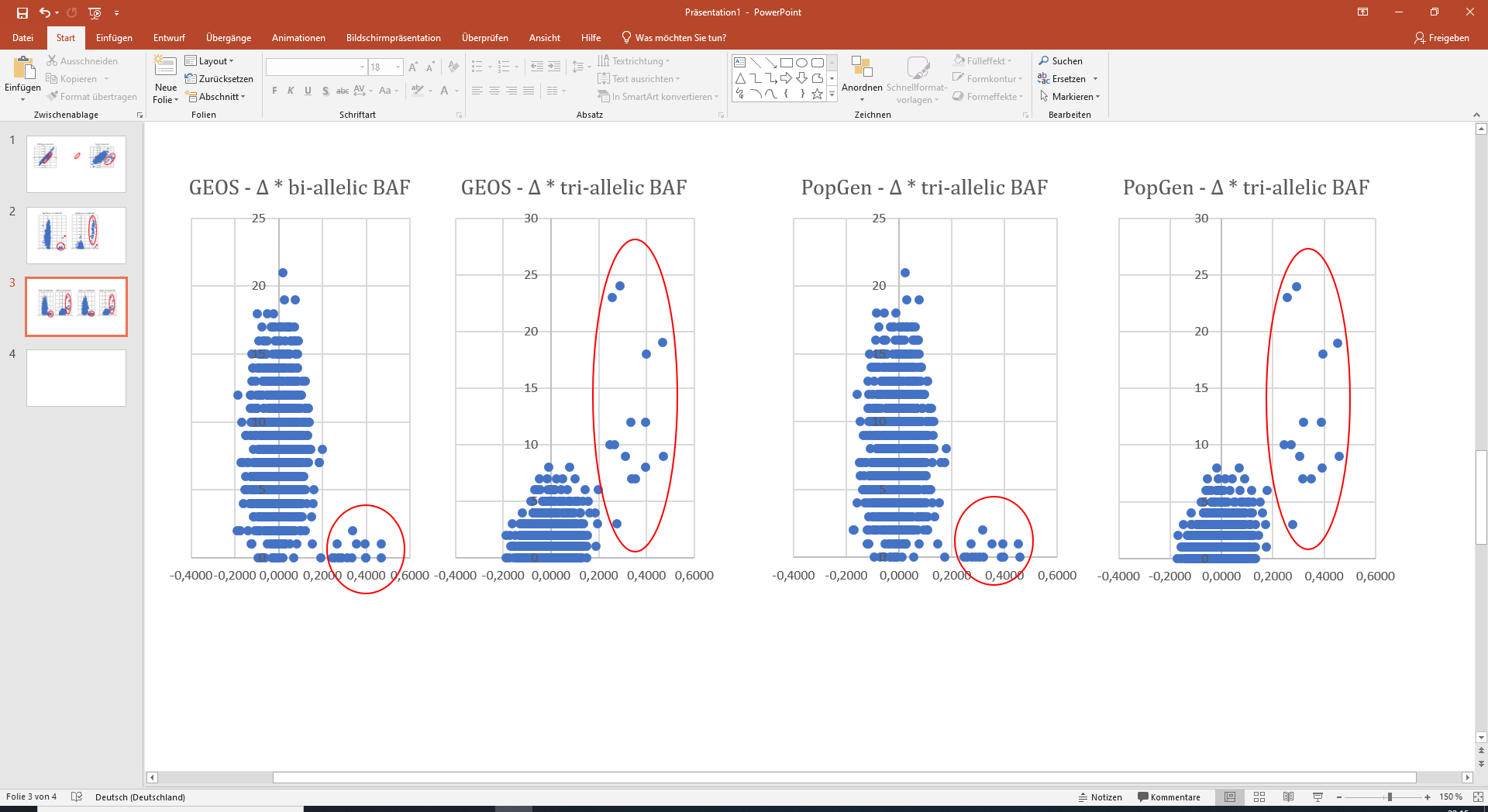


GEOS sample and PopGen Sampple: Scatterplots of Δ versus the count of bi-allelic (left panel) and tri-allelic (right panel) BAF-values. Legends similar as in the above Sign/Giscome scatterplot.

2. CNV-calling in genomic regions with clonal mosaicism


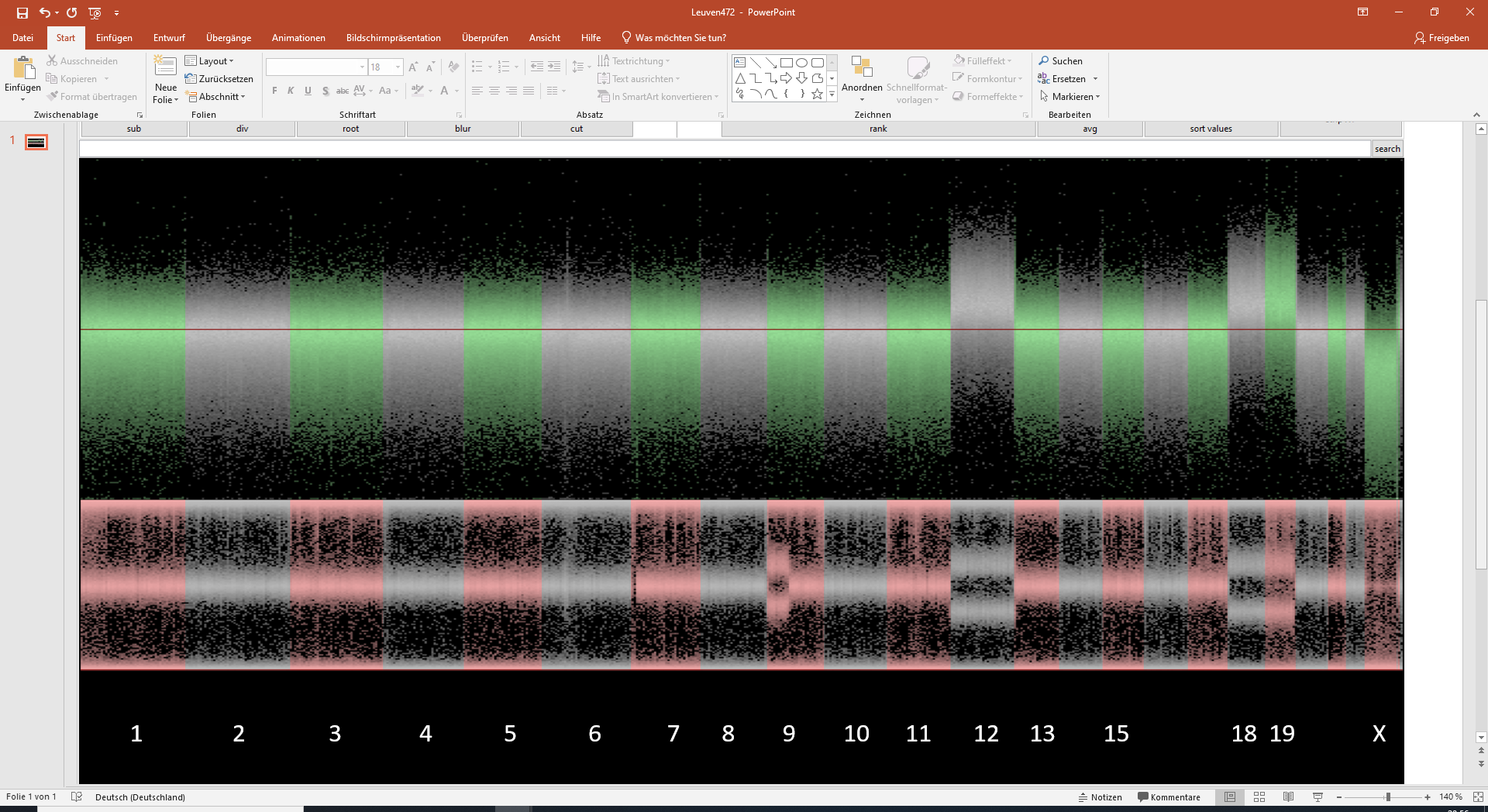


Visualization of a microarray sample genotyped on the Illumina 5M platform. LRR-values (upper panel) and BAF-values (lower panel) for all SNPs were plotted. Chromosomes were indicated. DNA was extracted from a venous blood puncture. White blood cells of this male patient (reduced LRR values and loss of heterozygosity of the X-chromosome) show trisomy for chromosomes 12, 18 and 19, inferred from the increased LRR values and tri-allelic BAF values. However, tri-allelic BAF values of chromosome 9p go together with normal (CN=2) LRR-values, indicating mosaicism.


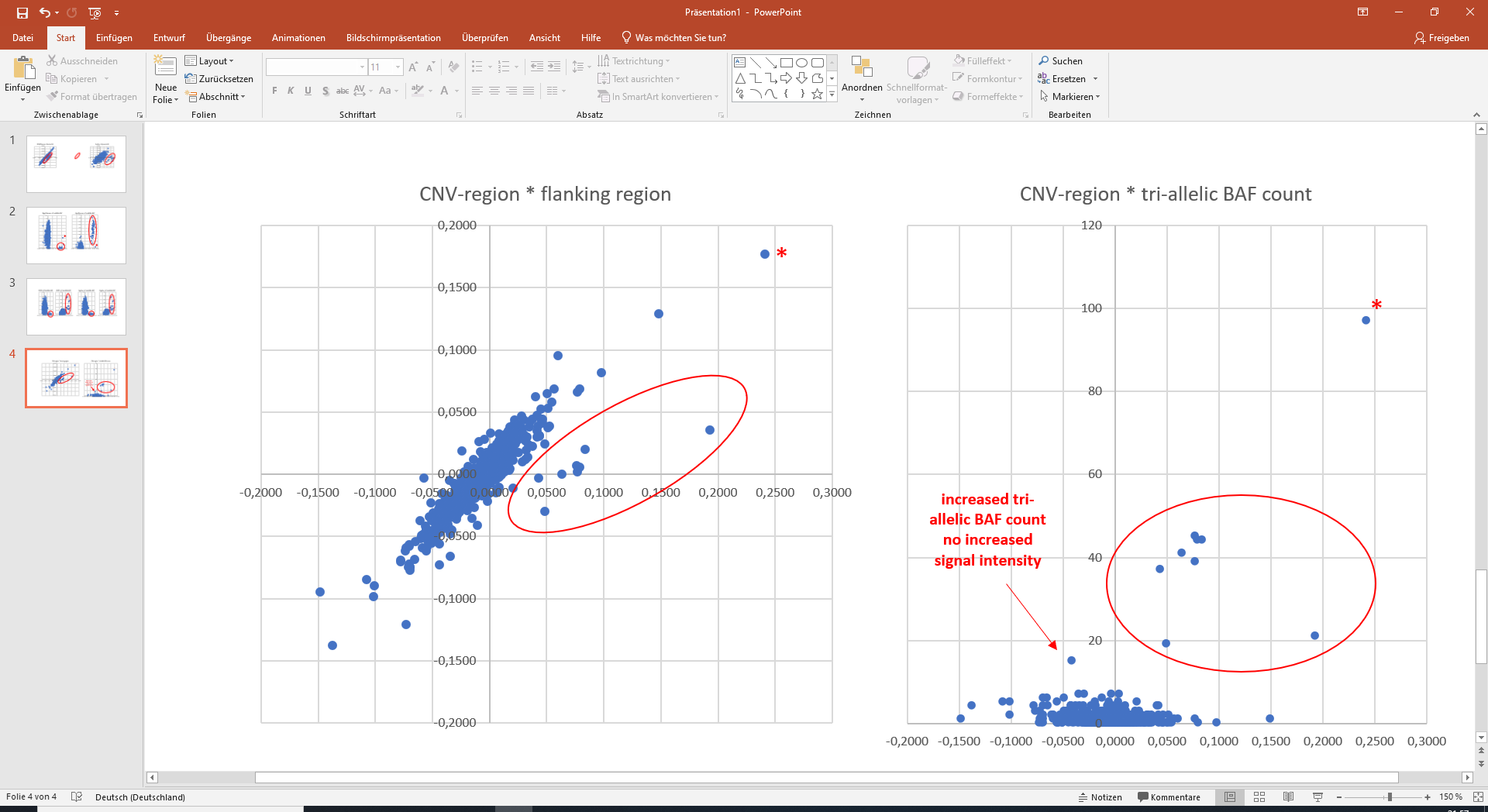


In the study population from Krakow, part of the SiGN/Giscome sample, we analyzed CNV in the tip of chromosome 9p. A scatterplot of CNV-region against flanking SNPs, find a somewhat irregular pattern of cases with increased CNV-region signals, compared to flanking signals. All cases have increased tri-allelic BAF counts, indicating that these findings are true duplications. Visual inspection of these cases after noise reduction with the noise-free-CNV software confirmed these findings. A larger duplication, extending into the selected flanking region, was observed in one individual, marked with the red star. Whereas all individuals with increased tri-allelic BAF counts have high signal intensities in the CNV target region, as expected for duplications, a single case (indicated with an arrow) has increased tri-allelic BAF counts, combined with “normal” LRR-values. Visual inspection of this case was shown before and revealed mosaicism.
